# CAR-T细胞治疗后第二原发肿瘤发生风险与管控策略

**DOI:** 10.3760/cma.j.cn121090-20260118-00036

**Published:** 2026-08

**Authors:** 若难 李, 文燕 王, 斐 杨, 乐乐 张, 均 施

**Affiliations:** 1 中国医学科学院血液病医院（中国医学科学院血液学研究所），血液与健康全国重点实验室，血液系统疾病国家临床医学研究中心，细胞生态海河实验室，天津 300020 State Key Laboratory of Experimental Hematology, National Clinical Research Center for Blood Diseases, Haihe Laboratory of Cell Ecosystem, Institute of Hematology & Blood Diseases Hospital, Chinese Academy of Medical Sciences & Peking Union Medical College, Tianjin 300020, China; 2 天津医学健康研究院，天津 301600 Tianjin Institutes of Health Science, Tianjin 301600, China

## Abstract

嵌合抗原受体T（CAR-T）细胞治疗在难治性血液系统肿瘤中取得了突破性进展，但其长期安全性日益受到重视，CAR-T细胞治疗后继发肿瘤（第二原发肿瘤，SPM）成为临床与基础研究的焦点。现有研究显示，CAR-T细胞治疗后不同类型SPM发生风险存在差异，2年累计发生率为0.08％～8％，中位发生时间为9.7～26.4个月。治疗相关骨髓增生异常综合征、急性髓系白血病等髓系恶性肿瘤为SPM主要类型，此外还包括实体瘤及少数T细胞淋巴瘤；仅在极少数T细胞淋巴瘤病例中检测到CAR转基因。其发生机制涉及病毒载体相关基因组整合风险、既往化疗或造血干细胞移植导致的遗传毒性损伤，以及免疫失衡和克隆性造血扩增等因素。未来研究可聚焦于开发更安全的基因编辑技术、将CAR-T细胞治疗前移以减少前期治疗的累积毒性，开展治疗前的遗传风险筛查以及治疗后的长期系统性监测，完善全流程管理将SPM发生风险控制在最低水平，从而在保证疗效的同时提升CAR-T细胞治疗的长期安全性。

## Abstract (en)

Chimeric antigen receptor T（CAR-T）cell therapy has revolutionized the treatment of refractory hematologic malignancies, but its long-term safety has become an increasing concern. Secondary primary malignancies（SPM）following CAR-T-cell therapy have emerged as a growing focus of clinical and translational research. Evidence to date suggests that the incidence of different types of SPMs vary, with a two-year cumulative incidence ranging from approximately 0.08％ to 8％ and a median time to onset of 9.7 to 26.4 months, predominantly involving myeloid malignancies such as myelodysplastic syndromes and acute myeloid leukemia, while solid tumors and rare T-cell lymphomas have also been reported. CAR transgenes have been detected in only a minority of T-cell lymphoma cases. The mechanisms involved vector-related genomic integration risks, genotoxic stress from prior chemotherapy or hematopoietic stem cell transplantation, immune dysregulation, and clonal hematopoietic expansion. Future research may focus on developing safer gene-editing technologies, moving CAR-T-cell therapy to earlier treatment lines to reduce cumulative toxicity from prior therapies, implementing genetic risk screening before treatment, and establishing long-term systematic monitoring after therapy. By optimizing full-process management, the risk of SPM can be minimized, thereby improving the long-term safety of CAR-T-cell therapy while maintaining its therapeutic efficacy.

嵌合抗原受体T（CAR-T）细胞治疗的问世标志着血液肿瘤免疫治疗迈进新纪元。自2017年获批以来，CAR-T细胞治疗已在复发/难治B细胞淋巴瘤、多发性骨髓瘤（MM）、急性淋巴细胞白血病等血液系统恶性肿瘤中取得显著疗效，部分患者可实现完全缓解[1]。其应用正逐步拓展至实体瘤及自身免疫性疾病等领域，早期研究表明经过工程化改造的T细胞通过靶向自身反应性B细胞，有望促使自身免疫性疾病达到长期缓解[2]–[4]。对于其引发的短期不良反应，如细胞因子释放综合征（CRS）以及免疫效应细胞活化相关神经毒性综合征（ICANS）的预防及治疗逐渐成熟，而长期不良反应即第二原发肿瘤（SPM）日益引发监管部门与学术界的共同警觉。

SPM指患者既往恶性肿瘤治疗后新发生的、不同于原发肿瘤且具有独立生物学起源的恶性肿瘤，不包括原发肿瘤的复发、转移或疾病进展。2023年11月，美国食品药品监督管理局（FDA）对接受CAR-T细胞治疗后出现SPM的患者展开调查[5]，指出CAR转导与T淋巴细胞恶性转化之间可能存在关联，并于2024年1月要求对已获批的6款CAR-T细胞治疗产品（tisagenlecleucel、axicabtagene ciloleucel、brexucabtagene autoleucel、lisocabtagene maraleucel、idecabtagene vicleucel、ciltacabtagene autoleucel）加入黑框警告。截至2023年12月31日，FDA在27 000多例接受CAR-T细胞治疗的患者中发现22例（约0.08％）继发T细胞淋巴瘤（TCL），其中仅3例（约0.01％）检测到CAR基因，提示CAR-T细胞产品可能与TCL发生相关[6]。截至2025年9月，全球共有14款靶向CD19或B细胞成熟抗原（BCMA）的CAR-T细胞产品获批上市，随着其应用拓展至实体瘤、自身免疫性疾病等领域，受益人群更加年轻化，任何低概率的致癌事件都有可能成为严峻的公共卫生问题。因此，本综述将重点关注CAR-T细胞治疗后SPM的发生率、类型特征、基因与免疫学驱动机制以及SPM的防治与长期监管进展，并总结需进一步研究的重点领域，以期更好地理解与预判此类风险，提升CAR-T细胞治疗的长期安全性。

一、CAR-T细胞治疗后SPM发生特征及临床类型

1. SPM发生情况：CAR-T细胞治疗后SPM总体发生率较低，目前报道其2年累计发生率为0.08％～8％，发生率从高到低依次为治疗相关髓系肿瘤（t-MN）、实体肿瘤和TCL[7]–[8]。t-MN与实体瘤的中位发病时间分别为9.7个月和26.4个月，TCL中位发生时间均于产品回输后24个月内（1～19个月）。意大利血液学会（SIE）与斯坦福大学分别开展大样本、长周期的CAR-T细胞治疗后的SPM随访研究，对651例和724例接受CAR-T细胞治疗的患者进行随访，前者报告SPM发生率为4.3％，以血液系统恶性肿瘤为主，与既往大剂量化疗和自体造血干细胞移植后的继发肿瘤率一致；后者发现血液系统继发肿瘤的3年累计发生率为6.5％，其中仅1例死亡病例可能与CAR-T细胞治疗相关的免疫抑制有关[7],[9]。另一项回顾性分析进一步强调了t-MN、实体瘤及TCL的罕见性，中位随访时间为3年的抗CD19 CAR-T细胞治疗后t-MN发生率为8.2％、实体瘤的发生率为1.9％，且549例患者中仅发现1例TCL[10]。Ghilardi等[11]研究同样显示CAR-T细胞治疗后TCL发生风险较低，治疗的449例患者中位随访61个月后，16例（3.6％）患者出现SPM，仅观察到1例TCL。CAR-T细胞治疗后SPM仍为罕见事件，但其潜在风险应受到重视。

2. SPM类型：多项研究数据提示最令人担忧的2种SPM是t-MN和TCL[7]–[8],[12]。其中，t-MN由于具有侵袭性强、预后差以及与既往细胞毒性药物治疗、淋巴细胞清除性化疗等累积基因损伤相关，被认为是CAR-T细胞治疗后最具临床意义的血液系统SPM；而TCL因涉及基因修饰T细胞潜在的插入突变风险及CAR转基因相关致瘤可能性，受到监管机构和学界的高度关注。基于全球药物警戒数据库的实证研究，通过分析FDA不良事件报告系统和VigiBase数据库的607例SPM临床资料，发现骨髓增生异常综合征（MDS）和急性髓系白血病（AML）是最常见的血液系统SPM，而基底细胞癌和皮肤鳞状细胞癌是主要的实体瘤类型[13]。TCL虽受关注，但在SPM中占比不足3％。另外CAR-T细胞治疗后TCL、MDS等亚型的报告风险比（ROR）显著升高，但需注意混杂因素（如患者基础疾病、既往治疗）对结果的影响[8]。

（1）t-MN：多项大样本研究显示（表1），t-MN的发生率为2％～8％，其危险因素与既往大剂量化疗、放疗及造血干细胞移植造成的累积遗传毒性损伤密切相关；部分患者在CAR-T细胞治疗前已存在克隆性造血，且发生t-MN后常检测到TP53等高危基因异常，提示既往治疗选择压力可能促进预存异常造血克隆扩增并最终演变为t-MN。总体而言，现有证据更支持既往治疗累积损伤与克隆性造血演变是CAR-T细胞治疗后t-MN发生的主要基础，尚缺乏CAR-T细胞本身直接致癌的明确证据。

**表1:** CAR-T细胞治疗后发生治疗相关髓系肿瘤（t-MN）研究报道

研究来源	原发疾病类型	CAR-T细胞产品	研究总体例数	SPM例数（％）	t-MN类型及例数（％）
Jacobson等[14]	DLBCL	Axi-cel	1 297	50（3.9）	MDS 15（1.2）；MDS/MPN 4（0.3）
Alkhateeb等[15]	B细胞NHL	多种	189	NR	MDS 8（4.2）；AML 2（1.1）
Jain等[16]	DLBCL	Axi-cel	275	23（8.4）	MDS 11（4.0）；AML 2（0.7）；CMML 1（0.4）
Neelapu等[17]	FL、MZL	Axi-cel	159	18（11.3）	MDS/AML 8（5.0）
Elsallab等[8]	B细胞NHL、B细胞ALL、MM	多种	12 394	536（4.3）	MDS 208（1.7）；AML 106（0.9）
Abramson等[18]	DLBCL	Liso-cel	270	22（8.1）	MDS 8（3.0）；AML 2（0.7）
Melody等[10]	B细胞NHL及其他	多种	582	45（7.7）	MDS 18（3.1）；AML 6（1.0）
Ghilardi等[11]	B细胞NHL、B细胞ALL、MM	多种	449	16（3.6）	MDS 2（0.4）；AML 1（0.2）
Cliff等[19]	多种	B细胞淋巴瘤	1 542	NR	MDS 40（2.6）；AML 14（0.9）
Tix等[20]	B细胞NHL、MM	多种	5 517	326（5.9）	血液系统恶性肿瘤121（2.2）
Kharfan-Dabaja等[21]	B细胞NHL	多种	88	NR	AML 1（1.1）
Jadlowsky等[22]	多种	多种	783	18（2.3）	MDS 2（0.3）；AML 3（0.4）；t-MN 10（1.3）
Farina等[23]	B细胞NHL、B细胞ALL	多种	555	14（2.5）	MDS 13（2.3）；AML 1（0.2）
Gazeau等[24]	多种	多种	539	29（5.4）	t-AML 11（2.0）；t-MDS 18（3.3）

**注** CAR-T细胞：嵌合抗原受体T细胞；SPM：第二原发肿瘤；DLBCL：弥漫大B细胞淋巴瘤；NHL：非霍奇金淋巴瘤；FL：滤泡性淋巴瘤；MZL：边缘区淋巴瘤；ALL：急性淋巴细胞白血病；MM：多发性骨髓瘤；Axi-cel：axicabtagene ciloleucel；Liso-cel：lisocabtagene maraleucel；NR：未报告；MDS：骨髓增生异常综合征；MPN：骨髓增殖性肿瘤；AML：急性髓系白血病；CMML：慢性粒-单核细胞白血病；t-AML：治疗相关AML；t-MDS：治疗相关MDS

（2）继发性TCL：如表2所示，CAR-T细胞治疗后继发性TCL发生率低，多数大样本研究显示其发生率约0.1％。已报道的病例主要与转基因阳性T细胞克隆扩增相关，如慢病毒或转座子载体插入诱变，也有部分在CAR构建过程中即存在潜在恶性克隆。大规模随访研究表明，CAR-T细胞治疗后发生TCL的风险明显低于t-MN，且多为个例。尽管如此，其潜在机制仍需进一步验证，未来应在CAR-T细胞生产环节加强基因整合位点检测，并结合长期随访监测，以便及早识别高危患者，由此降低相应临床风险。

**表2:** CAR-T细胞治疗后发生T细胞肿瘤（TCL）研究报道

研究来源	原发疾病类型	CAR-T细胞产品	研究总体例数	TCL例数（％）
Verdun等[6]	多种	多种	>27 000	22（约0.08）
Willyard[25]	多种	多种	约30 000	33（约0.11）
Ghilardi等[11]	B细胞NHL、B细胞ALL、MM	多种	449	1（0.22）
Abramson等[18]	DLBCL	Liso-cel	270	1（0.37）
Melody等[10]	多种	多种	582	1（0.17）
Cliff等[19]	多种	多种	1 542	1（0.06）
Jadlowsky等[22]	多种	多种	783	1（0.13）
Harrison等[26]	MM	Cilta-cel	208	2（0.96）
Dulery等[27]	多种	多种	3 066	1（0.03）

**注** CAR-T细胞：嵌合抗原受体T细胞；NHL：非霍奇金淋巴瘤；ALL：急性淋巴细胞白血病；DLBCL：弥漫大B细胞淋巴瘤；MM：多发性骨髓瘤；Liso-cel：lisocabtagene maraleucel；Cilta-cel：ciltacabtagene autoleucel

（3）继发性实体肿瘤：如表3所示，CAR-T细胞治疗后继发实体肿瘤累计发生率为1％～3％，最常见为皮肤癌，其次是泌尿生殖系统、消化系统及呼吸系统肿瘤。肿瘤组织未检测到CAR转基因，输注前后血液样本亦未检测到慢病毒复制，提示CAR-T细胞产品本身并未导致实体瘤的发生。

**表3:** CAR-T细胞治疗后发生实体肿瘤研究报道

研究来源	原发疾病类型	CAR-T细胞产品	研究总体例数	SPM例数（％）	实体肿瘤例数（％）
Pasquini等[28]	B细胞ALL、B细胞NHL	Tisagenlecleucel	410	12（2.9）	5（1.2）
Spiegel[29]	DLBCL	Axi-cel	275	21（7.6）	7（2.5）
Westin等[30]	DLBCL	Axi-cel	170	8（4.7）	5（2.9）
Lin等[31]^a^	MM	Ide-cel	62	8（12.9）	7（11.2）
Dreyling等[32]	FL	Tisagenlecleucel	97	3（3.1）	3（3.1）
Melody等[10]	B细胞NHL及其他	多种	582	45（7.7）	11（1.9）
Berning等[33]	DLBCL	多种	172	1（0.6）	1（0.6）

**注** CAR-T细胞：嵌合抗原受体T细胞；SPM：第二原发肿瘤；ALL：急性淋巴细胞白血病；NHL：非霍奇金淋巴瘤；DLBCL：弥漫大B细胞淋巴瘤；MM：多发性骨髓瘤；FL：滤泡性淋巴瘤；Axi-cel：axicabtagene ciloleucel；Ide-cel：idecabtagene vicleucel；^a^：引用内容为小样本早期研究结果，不宜外推至商业化产品总体风险水平

3. SPM发生的危险因素及临床风险特征：一项回顾性研究指出，不同研究报告的SPM发生率差异可能与治疗环境、随访时长及既往治疗线数不同有关，与CAR-T细胞产品类型（如Cilta-cel、Ide-cel等）相关性的差异无统计学意义；并且与标准治疗相比，CAR-T细胞治疗并未显著增加SPM发生风险（5.0％对4.9％）[20]，提示CAR-T细胞治疗后SPM的发生可能更多受到患者基础疾病特征及既往治疗暴露等因素影响。

SPM发生风险及时间特征可能与患者基础特征、既往治疗暴露及治疗相关因素有关，其中年龄、基线克隆性造血状态、既往治疗线数以及淋巴细胞清除方案可能影响SPM的发生模式。患者基线克隆性状态可能影响SPM发生风险，已有研究提示，部分CAR-T细胞治疗后发生持续性血细胞减少或继发髓系肿瘤的患者，在治疗前已存在克隆性造血，提示预存异常克隆可能是潜在风险因素[34]。其次患者年龄可能影响SPM的发生时间，年龄<40岁的患者在接受CAR-T细胞治疗后出现SPM的时间较老年患者更早[35]。这一发现具有重要的临床指导意义，提示在进行CAR-T细胞治疗后的随访监测时需要根据患者年龄采取不同的筛查策略和监测频率，为实现个体化的SPM预防提供了重要依据。治疗相关因素尤其是淋巴细胞清除性化疗，也可能影响SPM发生风险。虽然尚未有研究直接探索氟达拉滨或环磷酰胺给药与CAR-T细胞治疗后患者发生SPM之间的关系，但既往结果提示，这些化疗药物产生的致癌作用可能参与SPM发生，特别是在治疗相关的MDS/AML（t-MDS/AML）中更为明显[36]。

二、SPM的基因与免疫学驱动机制

现有研究提示，CAR-T细胞治疗后SPM并非由单一因素驱动，而是由载体相关遗传事件、治疗相关基因损伤以及免疫生态改变等多种机制共同作用。其中，少数CAR转基因阳性肿瘤可能与载体整合相关，而多数SPM则可能源于既往治疗导致的基因损伤、克隆选择及免疫调控异常。

1. CAR转基因相关基因组整合与插入突变风险：病毒构建体的整合通常发生在T细胞基因组内的活跃基因位点，如插入位点位于与癌症发生相关的DNA序列周围，则可能诱发癌症，提示插入诱变可能与转基因阳性肿瘤相关。截至2024年4月，德国国家药品安全监管机构保罗·埃利希研究所（PEI）共收到4例疑似与插入突变相关的病例报告[37]。在接受Cilta-cel治疗MM的患者中，已报道2例插入诱变导致的转基因阳性SPM病例[9],[38]。此外，一项使用piggyBac转座子系统传递CAR转基因的研究发现10例患者中2例发生淋巴瘤，其恶性T细胞的标志是转座子DNA[39]，均提示基因整合所致的插入突变是SPM发生的潜在原因之一。

2. 治疗前的遗传易感性与潜在恶性克隆：TP53异常在t-MN中较为常见，携带TP53异常的细胞克隆可能在化疗等治疗压力下获得选择优势并扩增，尤其是在既往接受多线治疗的年轻患者中，可能增加SPM发生风险。此外，细胞因子信号转导抑制蛋白1（SOCS1）突变可能通过增强CAR-T细胞活化、促进异常克隆扩增，或与CAR载体整合事件共同作用，参与CAR转基因阳性TCL的发生[40]。另外Harrison等[41]报告了1例淋巴结活检为CAR转基因阳性TCL，认为这种罕见肿瘤可能与核因子κB2（NFKB2）、甲基胞嘧啶双加氧酶2（TET2）或蛋白酪氨酸磷酸酶受体型B（PTPRB）的体细胞变异相关，且这些具有恶性潜能的变异可能在Cilta-cel制备前就已存在，提示治疗前潜在遗传异常可能是CAR-T细胞治疗后SPM发生的重要基础。

3. 免疫失衡与克隆选择压力：CAR-T细胞治疗伴随的免疫抑制状态削弱了对克隆造血的监视，从而促进SPM的发生。Vainstein等[42]报道4例MM患者在CAR-T细胞治疗后被诊断为MDS，检测到的MDS相关突变在治疗前均已以较低频率存在，提示出现的继发性MDS是由体内已存在的克隆造血在“免疫空窗期”快速扩增所致。有研究报道SPM的发病可能与抗原驱使的T细胞过度增殖有关。基因修饰后CAR持续刺激T细胞，且随着时间的推移，可增加累积突变的风险[9],[41]。CAR-T细胞本身可能会发展为TCL，患者在治疗前检测到癌前T细胞克隆时SPM发生风险可能更高。同样，Hamilton团队的研究结果表明，EB病毒阳性淋巴瘤可能更易由CAR-T细胞治疗后产生的克隆性造血引发[9]。

三、SPM的防治与管理策略进展

目前针对CAR-T细胞治疗后SPM的有效治疗策略仍不明确[43]。近期有研究报道1例CAR-T细胞治疗继发的CAR转基因阳性外周TCL患者治疗取得成功：该例患者因MM接受BCMA CAR-T细胞治疗后6个月发病，单细胞测序提示肿瘤细胞CCR4高表达；经药物筛选后，患者接受抗CCR4抗体莫格利珠单抗+脂质体多柔比星+吉西他滨联合方案治疗获得完全缓解并在10个月随访期间无复发[44]。以上提示CCR4可能成为CAR相关毒性效应（包括外周TCL尤其是皮肤受累患者）的潜在治疗靶点，但仍需更多临床与机制研究加以验证。

现有研究证据支持将CAR-T细胞治疗前移的策略，既可降低患者在既往多线化疗中累积的遗传毒性损伤风险，且不会增加SPM发生风险[45]–[46]。因此，CAR-T细胞治疗后SPM的防控可从以下3个方面入手。

1. 源头干预与制造工艺优化：一级预防是从生产与工艺源头降低SPM的发生风险，其核心在于减少整合相关诱变与基因毒性风险。首先，定点整合与“基因组安全港”策略被认为是降低载体整合风险的关键方向。传统γ-逆转录病毒与慢病毒载体多呈半随机整合特征，可能在特定情境下引发LMO2等原癌基因异常激活或TP53等抑癌基因功能受损，从而增加插入突变相关风险[47]。近年来，借助成簇规律间隔短回文重复序列及其相关蛋白9（CRISPR-Cas9）、转录激活因子样效应物核酸酶（TALEN）等基因编辑工具，通过同源定向修复（HDR）将CAR转基因精准导入已验证的基因组安全港或功能位点，如腺相关病毒整合位点1（AAVS1）、T细胞受体α恒定区（TRAC）、CCR5、柠苹酰-CoA裂合酶（CLYBL），有望在不削弱抗肿瘤效应的前提下，显著降低随机整合带来的插入突变风险[47]–[48]。此外，采用以Sleeping Beauty、piggyBac转座子为代表的非病毒基因转移体系或闭环线性双链DNA（dsDNA）等替代平台，可减少病毒相关序列及其整合偏好性带来的潜在影响，从而在一定程度上降低克隆优势、异常克隆扩增等风险[47]。

其次，应进一步优化起始T细胞亚群与体外培养工艺。通过富集具有更高增殖潜能与长期持久性的T细胞亚群，并在满足制备需求的前提下缩短体外扩增周期、优化细胞因子组合与培养条件，可降低长期体外培养相关的基因组不稳定性与表观遗传漂变风险，从而提升产品一致性与长期安全性。

2. 风险分层与高危人群筛查：二级预防聚焦治疗前对易感人群的识别及易感状态的干预。克隆性造血的基线筛查被认为是目前循证证据相对充分的二级预防措施之一。现有研究提示，30％～40％的CAR-T细胞治疗候选患者，尤其是高龄及多线治疗患者，可检测到克隆性造血相关体细胞突变，常见基因为DNA甲基转移酶3A（DNMT3A）、TET2、Mg²⁺/Mn²⁺依赖性蛋白磷酸酶1D（PPM1D）与ASXL转录调节因子1（ASXL1）等。该类克隆在氟达拉滨/环磷酰胺清淋化疗等治疗相关选择压力作用下，可能发生优势扩增，并与后续t-MN以及迁延性血细胞减少发生风险升高相关[9],[48]。尽管目前尚缺乏强制性、统一的筛查指南，多数专家共识建议：对存在基线血细胞减少、或既往接受过烷化剂治疗/放疗等暴露史的高危患者，可基于外周血或骨髓样本开展靶向二代测序，并对克隆性造血相关突变进行等位基因频率量化，以用于风险分层与随访方案优化。需要强调的是，筛查阳性结果通常不应作为挽救性CAR-T细胞治疗的禁忌证，而是作为加强血液学监测与并发症预警的依据。此外，遗传易感性评估在早发肿瘤或多原发癌家族史患者中具有提示价值，尤其是涉及DNA损伤修复通路的胚系突变携带者，其CAR-T细胞治疗后SPM发生风险阈值可能更低[49]。

3. 长期随访与风险导向监测标准化：三级预防的核心在于通过制度化随访实现SPM的早发现、早诊断与早干预，从而降低相关死亡风险。

（1）全程风险导向监测与早期识别：建议在CAR-T细胞输注后2年内每6～12个月进行系统评估，包括外周血细胞计数、血清蛋白电泳、乳酸脱氢酶及必要的影像学检查。若出现原因不明的血细胞减少、T细胞受体（TCR）克隆性重排，或外周血CAR转基因持续阳性等提示信号，应提高警惕并升级评估，可结合骨髓穿刺或活检与PET-CT等检查，以排除隐匿性髓系肿瘤或TCL。

（2）建立标准化报告体系与登记系统：鉴于SPM的罕见性，单一中心难以获得有效样本量，建议强制或半强制化向国际血液与骨髓移植研究中心或FDA不良事件报告系统等公共数据库上报SPM病例，并明确区分以下3种克隆归属状态：与原发肿瘤克隆相关（疾病进展）；与CAR-T细胞克隆相关（转基因阳性继发肿瘤）；与新发独立克隆相关（真性SPM）。克隆关系判定可结合整合位点分析、单细胞组学及TCR谱系追踪等方法进行综合验证。

（3）临床药学与多学科协作：临床药师应全程参与CAR-T细胞治疗药物警戒，建立SPM识别、分析、报告的药学服务路径，并纳入数据驱动的持续质量改进项目。

随着越来越多的患者接受CAR-T细胞治疗，了解和认识SPM至关重要。在接受治疗前筛查克隆性造血并在治疗后进行随访监测，可能有助于识别高危患者。未来研究应深入探讨载体整合、获得性遗传和表观遗传损伤、细胞因子驱动的增殖以及反复感染在驱动T细胞转化和淋巴瘤发生中的相互作用。并依据基因组表型和功能特征制定的有效治疗方案，改善患者预后。

四、总结

近年来，CAR-T细胞治疗在血液系统恶性肿瘤和自身免疫性疾病中取得了显著进展，新型in vivo CAR-T技术的不断涌现，有望显著降低CRS等不良反应[50]。随着CAR-T细胞治疗新技术层出不穷，临床应用不断扩大，其长期安全性问题备受瞩目。尽管SPM总体发生率较低，但其对患者生存和预后影响显著，因此需要建立完善的长期随访与监测体系。未来研究应从以下方面着手，以实现更安全、持久的治疗获益：首先，应建立更大规模、长期随访的真实世界队列，以明确不同CAR-T细胞产品、适应证及患者人群中SPM的真实发生率与风险因素。其次，深入解析CAR-T细胞治疗后SPM的分子机制，涵盖载体相关基因组整合与插入突变风险、治疗前克隆性造血及异常克隆演变，以及治疗后免疫失衡介导的克隆选择压力。同时，人类疱疹病毒6型（HHV-6）再激活相关免疫异常、端粒损伤及基因组不稳定性等潜在机制也值得进一步探索[51]。尽管这些新兴机制的临床意义尚需通过前瞻性队列及机制性研究加以验证，但相关研究将为识别高危人群、优化CAR-T细胞产品设计和制定个体化监测策略提供重要依据。第三，应实施系统性的监测和早期预警策略，如应用多组学检测、循环DNA监测及长期免疫学评估，从而实现对继发性恶性肿瘤的早期发现和干预。随着机制研究和技术改进的不断深入，未来有望更好地预防和管理CAR-T细胞治疗后SPM，为患者带来更安全和持久的临床获益。

另外有关CAR-T细胞治疗的伦理和规范监管问题备受关注，即使在2025年6月FDA取消了对相关CAR-T细胞产品使用环境、认证医疗机构、紧急配备医疗干预等的制度性限制，但黑框警告仍然存在。未来，随着CAR-T细胞治疗在自身免疫病等非肿瘤领域的拓展，这些伦理与监管挑战将更加复杂，如何在创新与规范之间取得动态平衡，将直接影响该治疗方案的可持续发展。

## References

[1] Cappell KM, Kochenderfer JN (2023). Long-term outcomes following CAR T cell therapy: what we know so far[J]. Nat Rev Clin Oncol.

[2] Wu D, Xu-Monette ZY, Zhou J (2025). CAR T-cell therapy in autoimmune diseases: a promising frontier on the horizon[J]. Front Immunol.

[3] Müller F, Taubmann J, Bucci L (2024). CD19 CAR T-cell therapy in autoimmune disease - a case series with follow-up[J]. N Engl J Med.

[4] Mougiakakos D, Krönke G, Völkl S (2021). CD19-targeted CAR T cells in refractory systemic lupus erythematosus[J]. N Engl J Med.

[5] Dolgin E (2024). Personalized DNA vaccine immunogenic against melanoma[J]. Cancer Discov.

[6] Verdun N, Marks P (2024). Secondary cancers after chimeric antigen receptor T-cell therapy[J]. N Engl J Med.

[7] Barone A, Chiappella A, Casadei B (2024). Secondary primary malignancies after CD-19 directed CAR-T-cell therapy in lymphomas: a report from the Italian CART-SIE study[J]. Br J Haematol.

[8] Elsallab M, Ellithi M, Lunning MA (2024). Second primary malignancies after commercial CAR T-cell therapy: analysis of the FDA Adverse Events Reporting System[J]. Blood.

[9] Hamilton MP, Sugio T, Noordenbos T (2024). Risk of second tumors and T-cell lymphoma after CAR T-cell therapy[J]. N Engl J Med.

[10] Melody M, Epperla N, Shouse G (2024). Subsequent malignant neoplasms in patients previously treated with anti-CD19 CAR T-cell therapy[J]. Blood Adv.

[11] Ghilardi G, Fraietta JA, Gerson JN (2024). T cell lymphoma and secondary primary malignancy risk after commercial CAR T cell therapy[J]. Nat Med.

[12] Hamilton MP, Alizadeh AA (2024). Rarity & risk of secondary hematologic neoplasms after CAR T-cell therapies[J]. Transplant Cell Ther.

[13] Zhao A, Zhao M, Qian W (2022). Secondary myeloid neoplasms after CD19 CAR T therapy in patients with refractory/relapsed B-cell lymphoma: case series and review of literature[J]. Front Immunol.

[14] Jacobson CA, Locke FL, Ma L (2022). Real-world evidence of axicabtagene ciloleucel for the treatment of large B cell lymphoma in the United States[J]. Transplant Cell Ther.

[15] Alkhateeb HB, Mohty R, Greipp P (2022). Therapy-related myeloid neoplasms following chimeric antigen receptor T-cell therapy for Non-Hodgkin Lymphoma[J]. Blood Cancer J.

[16] Jain MD, Spiegel JY, Nastoupil LJ (2024). Five-year follow-up of standard-of-care axicabtagene ciloleucel for large B-cell lymphoma: results from the US Lymphoma CAR T Consortium[J]. J Clin Oncol.

[17] Neelapu SS, Chavez JC, Sehgal AR (2024). Three-year follow-up analysis of axicabtagene ciloleucel in relapsed/refractory indolent non-Hodgkin lymphoma (ZUMA-5)[J]. Blood.

[18] Abramson JS, Palomba ML, Gordon LI (2024). Two-year follow-up of lisocabtagene maraleucel in relapsed or refractory large B-cell lymphoma in TRANSCEND NHL 001[J]. Blood.

[19] Cliff E R S, Lafeuille P, Diamond S (2024). Secondary hematologic malignancies in patients following chimeric antigen receptor T-cell therapy: aggregated clinical trial data from 1542 patients[J]. Blood.

[20] Tix T, Alhomoud M, Shouval R (2024). Second primary malignancies after CAR T-cell therapy: a systematic review and meta-analysis of 5,517 lymphoma and myeloma patients[J]. Clin Cancer Res.

[21] Kharfan-Dabaja MA, Mohty R, Easwar N (2025). Chimeric antigen receptor T cell therapy in octogenarians with B cell lymphoma: a real-world US multicenter collaborative study[J]. Bone Marrow Transplant.

[22] Jadlowsky JK, Hexner EO, Marshall A (2025). Long-term safety of lentiviral or gammaretroviral gene-modified T cell therapies[J]. Nat Med.

[23] Farina M, Bernardi S, Malagola M (2025). Real-world collection of secondary myeloid neoplasms after CD19 CAR-T cell therapy: first report of the ClonHema study[J]. Bone Marrow Transplant.

[24] Gazeau N, Beauvais D, Tilmont R (2025). Myeloid neoplasms after CD19-directed CAR T cells therapy in long-term B-cell lymphoma responders, a rising risk over time?[J]. Leukemia.

[25] Willyard C (2024). Do cutting-edge CAR-T-cell therapies cause cancer? What the data say[J]. Nature.

[26] Harrison SJ, Touzeau C, Kint N (2025). CAR+ T-cell lymphoma after Cilta-cel therapy for relapsed or refractory myeloma[J]. N Engl J Med.

[27] Dulery R, Guiraud V, Choquet S (2025). T cell malignancies after CAR T cell therapy in the DESCAR-T registry[J]. Nat Med.

[28] Pasquini MC, Hu ZH, Curran K (2020). Real-world evidence of tisagenlecleucel for pediatric acute lymphoblastic leukemia and non-Hodgkin lymphoma[J]. Blood Adv.

[29] Spiegel JY, Jain MD, Nastoupil LJ (2023). Five year outcomes of patients with large B-cell lymphoma treated with standard-of-care axicabtagene ciloleucel: results from the US Lymphoma CAR-T Cell Consortium[J]. Blood.

[30] Westin JR, Oluwole OO, Kersten MJ (2023). Survival with axicabtagene ciloleucel in large B-cell lymphoma[J]. N Engl J Med.

[31] Lin Y, Raje NS, Berdeja JG (2023). Idecabtagene vicleucel for relapsed and refractory multiple myeloma: post hoc 18-month follow-up of a phase 1 trial[J]. Nat Med.

[32] Dreyling M, Fowler NH, Dickinson M (2024). Durable response after tisagenlecleucel in adults with relapsed/refractory follicular lymphoma: ELARA trial update[J]. Blood.

[33] Berning P, Shumilov E, Maulhardt M (2024). Chimeric antigen receptor-T cell therapy shows similar efficacy and toxicity in patients with diffuse large B-cell lymphoma aged 70 and older compared to younger patients: a multicenter cohort study[J]. Hemasphere.

[34] Martino M, Porto G, Policastro G (2024). Effectiveness of CAR-T treatment toward the potential risk of second malignancies[J]. Front Immunol.

[35] Shen J, Hu R, Lin A (2024). Characterization of second primary malignancies post CAR T-cell therapy: real-world insights from the two global pharmacovigilance databases of FAERS and VigiBase[J]. EClinicalMedicine.

[36] Maurer K, Jacobson CA (2025). T-cell neoplasias and secondary malignancies after CAR-T cell therapy: current knowledge, risk factors, and implications from CAR-T engineering strategies[J]. Leuk Lymphoma.

[37] Berg P, Ruppert-Seipp G, Müller S (2025). CAR T-cell-associated secondary malignancies challenge current pharmacovigilance concepts[J]. EMBO Mol Med.

[38] Ozdemirli M, Loughney TM, Deniz E (2024). Indolent CD4+ CAR T-Cell Lymphoma after Cilta-cel CAR T-Cell Therapy[J]. N Engl J Med.

[39] Micklethwaite KP, Gowrishankar K, Gloss BS (2021). Investigation of product-derived lymphoma following infusion of piggyBac-modified CD19 chimeric antigen receptor T cells[J]. Blood.

[40] Perica K, Jain N, Scordo M (2025). CD4+ T-cell lymphoma harboring a chimeric antigen receptor integration in TP53[J]. N Engl J Med.

[41] Harrison SJ, Nguyen T, Rahman M (2023). CAR+ T-cell lymphoma post ciltacabtagene autoleucel therapy for relapsed refractory multiple myeloma[J]. Blood.

[42] Vainstein V, Avni B, Grisariu S (2023). Clonal myeloid dysplasia following CAR T-cell therapy: chicken or the egg?[J]. Cancers (Basel).

[43] Mitchell E, Vassiliou GS (2024). T-cell cancer after CAR T-cell therapy[J]. N Engl J Med.

[44] Aleman A, Van Oekelen O, Melnekoff DT (2025). Targeted therapy of CAR+ T-cell lymphoma after anti-BCMA CAR T-cell therapy[J]. N Engl J Med.

[45] Locke FL, Miklos DB, Jacobson CA (2022). Axicabtagene ciloleucel as second-line therapy for large B-cell lymphoma[J]. N Engl J Med.

[46] San-Miguel J, Dhakal B, Yong K (2023). Cilta-cel or standard care in lenalidomide-refractory multiple myeloma[J]. N Engl J Med.

[47] Safarzadeh Kozani P, Safarzadeh Kozani P (2025). Preventing secondary primary malignancies (SPMs) in CAR-T cell therapy through site-specific transgene integration into genomic safe harbors (GSHs)[J]. J Transl Med.

[48] Das DK (2025). Second primary cancers & CAR-T therapy: risks, insights & mitigation[J]. Oncol Times.

[49] Patel SA, Spiegel JY, Dahiya S (2025). Second primary cancer after chimeric antigen receptor-T-cell therapy: a review[J]. JAMA Oncol.

[50] Wang Q, Xiao ZX, Zheng X (2025). In vivo CD19 CAR T-cell therapy for refractory systemic lupus erythematosus[J]. N Engl J Med.

[51] Zhou Z, Zhang G, Xu Y (2024). The underlying mechanism of chimeric antigen receptor (CAR)-T cell therapy triggering secondary T-cell cancers: Mystery of the Sphinx?[J]. Cancer Lett.

